# Amyloid β peptide-mediated neurotoxicity is attenuated by the proliferating microglia more potently than by the quiescent phenotype

**DOI:** 10.1186/1423-0127-20-78

**Published:** 2013-10-23

**Authors:** Huey-Jen Tsay, Yung-Cheng Huang, Fong-Lee Huang, Chia-Ping Chen, Yu-Chun Tsai, Ying-Hsiu Wang, Mine-Fong Wu, Feng-Yi Chiang, Young-Ji Shiao

**Affiliations:** 1 Institute of Neuroscience, Brain Research Center, National Yang-Ming University, Taipei 11221, Taiwan; 2 Department of Physical Medicine and Rehabilitation, Cheng Hsin General Hospital, Taipei 11221, Taiwan; 3 Institute of Anatomy and Cell Biology, National Yang-Ming University, Taipei 11221, Taiwan; 4 Institute of Biopharmaceutical Science, National Yang-Ming University, Taipei 11221, Taiwan; 5 National Research Institute of Chinese Medicine, Taipei 11221, Taiwan, R.O.C; 6 Ph.D Program for the Clinical Drug Discovery from Botanical Herbs, College of Pharmacy, Taipei Medical University, Taipei 110, Taiwan, R.O.C

**Keywords:** Alzheimer’s disease, Microglia, Functional phenotypes, Neuroinflammation, Amyloid β protein, Neurotoxicity

## Abstract

**Background:**

The specific role of microglia on Aβ-mediated neurotoxicity is difficult to assign *in vivo* due to their complicated environment in the brain. Therefore, most of the current microglia-related studies employed the isolated microglia. However, the previous *in vitro* studies have suggested either beneficial or destructive function in microglia. Therefore, to investigate the phenotypes of the isolated microglia which exert activity of neuroprotective or destructive is required.

**Results:**

The present study investigates the phenotypes of isolated microglia on protecting neuron against Aβ-mediated neurotoxicity. Primary microglia were isolated from the mixed glia culture, and were further cultured to distinct phenotypes, designated as proliferating amoeboid microglia (PAM) and differentiated process-bearing microglia (DPM). Their inflammatory phenotypes, response to amyloid β (Aβ), and the beneficial or destructive effects on neurons were investigated. DPM may induce both direct neurotoxicity without exogenous stimulation and indirect neurotoxicity after Aβ activation. On the other hand, PAM attenuates Aβ-mediated neurotoxicity through Aβ phagocytosis and/or Aβ degradation.

**Conclusions:**

Our results suggest that the proliferating microglia, but not the differentiated microglia, protect neurons against Aβ-mediated neurotoxicity. This discovery may be helpful on the therapeutic investigation of Alzheimer’s disease.

## Background

Microglia originates from primitive progenitors derived from various sources and migrates into central nervous system (CNS) during early embryogenesis [1]. In the adult CNS, the number of microglia is hypothesized to be maintained via self-replication or by local progenitor cell division [2,3]. Local proliferation leads to an increased number of microglia in the spinal cord of postnatal rat [4]. In several neurodegenerative diseases, including Alzheimer’s disease (AD), an increased number of activated microglia is commonly observed near the foci of lesions [5-7]. Therefore, it has been proposed that microglial proliferation near lesion foci is critical for restoring homeostasis and inducing consequent CNS repair [8].

The proliferation and differentiation of microglia responding to the intrinsic and extrinsic stimuli depend on a set of growth factors, such as macrophage-colony stimulating factor (M-CSF) [9,10]. Moreover, distinct phenotypic microglia has been identified to exist in specific brain regions [11,12]. Microglia may exert both protective and pathogenic functions in the CNS [13-15]. Microglia-mediated inflammation will impede the treatment of CNS diseases. Therefore, it is essential to identify mechanisms of activating specific microglial phenotypes that contribute to neurodegeneration to define appropriate therapeutic targets aimed at modulating microglia activities [16].

Despite microglia play in the maintenance of CNS homeostasis and AD pathogenesis [7,17], our understanding of the molecular mechanisms responsible for microglia development and function is still fragmented. Microglia may respond to minor stimuli, which is a major impediment for the cellular characterization of microglia *in vivo*[18]. Isolation of amoeboid microglia from mixed glial cultures is a reliable method to verify the distinct populations of microglial phenotypes in a mixed glial culture [19,20]. In mixed glial culture, the amoeboid cells typically rest on top of the astrocyte monolayer and can appear as proliferating cell clusters promoted by astrocyte-secreted mitogens [21]. Ramified microglia was underneath the astrocyte layer, and the numbers of ramified cells increase with time in culture. The ramified population has decreased phagocytic and proliferation abilities.

Macrophage activation states have been classified as ‘classical’ activation and ‘alternative’ activation [22-24]. In general, classically activated macrophages are most commonly associated with pathogenic disease states [25]. In contrast, alternative activation states are generally associated with protection from diseases. In spite of the accepted concept that microglia are brain-resident macrophages, microglia exhibit several morphological features that distinguish them from macrophages. These include their ramified branches in the steady-state phenotype and the amoeboid morphology of their renewed and activated phenotypes [1,3,26,27]. Nevertheless, the classical or alternative activation concept of macrophages may be adapted to microglia at their steady-state and activated status [28-31]. Steady-state microglia exhibits a resting-like phenotype, characterized morphologically by extensively ramified processes that continuously monitor their surroundings in the CNS [32]. The property of the activated amoeboid microglia is similar to macrophages [33]. However, the primary determinants of ramified phenotype and amoeboid phenotype of microglia under pathological conditions in CNS are less well defined and may be different from those of macrophages in peripheral tissues.

AD is one of the most common age-dependent neurodegenerative diseases. AD pathology is characterized by the accumulation of amyloid-β (Aβ) containing neuritic plaques [34]. In addition to their direct neurotoxicity, both oligomeric Aβ (oAβ) and fibrillary Aβ (fAβ) are known to activate microglia [35-37]. Aβ-activated microglia produces pro-inflammatory mediators, which are neurotoxic [14]. It has been suggested that microglia can be neuroprotective by clearing Aβ species. Although the number of microglia is increased at the vicinity of senile plaques, the microglial phenotype responsible for pathological inflammation or neuroprotection remains to be verified.

In this study, distinct effect of the proliferating and differentiated microglia in the neuronal survival was observed *in vitro.* The proliferating amoeboid microglia (PAM) and the differentiated process-bearing microglia (DPM) had different responses to neurons or Aβ-mediated neurotoxicity. Their ability to protect neurons against Aβ was determined by co-culturing microglia with cortical neurons. In summary, we have cultured two microglia phenotypes from the same lineage, which contribute to the beneficial or destructive effects on neurons.

## Methods

### Materials

Mouse monoclonal antibody to GFAP and Hoechst 33258 were purchased from Invitrogen (Carlsbad, CA, USA). An enhanced chemiluminescence detection reagent and anti-rabbit IgG antibody conjugated with horseradish peroxidase were obtained from GE Healthcare (Buckinghamshire, UK). Donkey anti-goat IgG antibody conjugated with cy5, and donkey anti-rabbit or anti-mouse IgG antibodies conjugated with fluorescein were obtained from Jackson ImmunoResearch (West Grove, PA, USA). Rabbit polyclonal anti-Iba-1 antibody was purchased from Abcam (Cambridge, MA, USA). Mouse monoclonal anti-CD11b and goat polyclonal anti-tau antibodies were from Santa Cruz Biotechnology (Santa Cruz, CA, USA). Mouse anti-CD11b monoclonal antibody (OX-42 clone) was from Serotec (Kidlington, Oxford, UK). Mouse anti-CD68, anti-β-actin monoclonal antibodies, and Aβ1-42 were purchased from Millipore (Billerica, MA, USA). The TUNEL assay kit was obtained from Calbiochem (Darmstadt, Germany). Aβ25-35, lipopolysaccharide (LPS), and polyclonal anti-Aβ antibody were from Sigma (St. Louis, MO, USA). Recombinant rat interferon-γ (IFN-γ) was from PeproTech (London, UK). The immunoassay kits for interleukine-1β (IL-1β) and tumor necrosis factor-α (TNF-α) were from R&D Systems (Minneapolis, MN, USA).

### Preparation and biochemical characterization of Aβ25-35, oAβ and fAβ

fAβ25-35 was prepared by dissolving Aβ25-35 in H_2_O at 1 mM and aging for 1 week at 37°C. oAβ and fAβ were prepared as described [38,39]. A diluted solution of Aβs was spotted onto a mica slide and scanned using an Agilent® 5400 atomic force microscope (Molecular Imaging Corporation, Tempe, AZ) as described previously [20,40].

### Cell cultures

LADMAC cells (ATCC, CRL-2420) secrete M-CSF capable of supporting the *in vitro* proliferation of bone marrow macrophages [41]. LADMAC cells were cultured in Minimal Essential Medium (MEM) containing 10% fetal bovine serum (FBS). Once they had reached confluence, the cells were cultured for 7 days without changing the medium. Thereafter, the conditioned medium was used to culture DPM microglia.

Primary cultures of neonatal cortical microglia were prepared from the cerebral cortex of Sprague Dawley rat pups at postnatal day 5 [19,20]. Briefly, 5-day-old pups were anesthetized with ether and sacrificed by decapitation. Primary mixed glial cells were prepared from the cerebral cortex and maintained in DMEM/F12 medium containing 10% FBS for 5 days. The medium was replaced with fresh culture medium and incubated for 1 day. PAM was isolated from mixed glial cultures by shaking at 75 rpm overnight on an orbital shaker. Thereafter, cells were cultured in Neurobasal medium/B27 supplement for 1 day. Otherwise, PAM were proliferated in DMEM containing 10% FBS and 20% LADMAC-conditioned medium for 5 days, and then the cells were stripped and cultured in Neurobasal medium/B27 supplement for 1 day (i.e., DPM).

Primary cultures of neonatal cortical neurons were prepared from the cerebral cortex of Sprague Dawley rat pups at postnatal day 1 [42]. Briefly, pups were anesthetized with ether and sacrificed by decapitation. The cortex was digested in 0.5 mg/ml papain at 37°C for 15 min and dissociated in Hibernate A medium (containing B27 supplement) by trituration. Cells were plated (5 × 10^4^ cells/cm^2^) onto poly-L-lysine-coated plates and maintained in Neurobasal medium containing B27 supplement, 10 units/ml penicillin, 10 μg/ml streptomycin, and 0.5 μg/ml glutamine (5% CO_2_/95% O_2_) for 3 days. Cells were then exposed to cytosine-β-D arabinofuranoside (5 μM) for 1 day to eliminate the proliferation of non-neuronal cells.

### Immunocytochemistry

Treated cells were fixed with 4% paraformaldehyde (in PBS) at room temperature for 15 min and permeabilized with 0.5% Triton X-100 (in PBS) for 10 min. Cells were blocked with 10% normal donkey serum (in PBS containing 0.5% BSA) at room temperature for 2 h. Cells were treated to detect microglia using goat anti-Iba-1, rabbit anti-CD11b, and mouse anti-CD68 antibody. Cortical neuron morphology was assessed using mouse anti-microtubule associated protein 2 (MAP2) antibody and goat anti-tau antibody. Donkey anti-rabbit IgG or anti-goat IgG conjugated with cy5 and donkey anti-mouse IgG conjugated with fluorescein were used as secondary antibodies. For the study of Aβ phagocytosis, anti-Aβ1-40 polyclonal antibody and donkey anti-rabbit IgG antibody conjugated with fluorescein were used as primary and secondary antibodies, respectively.

### Measurement of nitrite and cytokines

Nitrite content (nitric oxide release) was measured by incubating culture medium with an equal volume of Griess reagent (0.05% N-(1-naphthyl)-ethylene-diamine dihydrochloride, 0.5% sulfanilamide, and 1.25% phosphoric acid). After incubation, the optical density was detected at a wavelength of 540 nm using a microplate reader with NaNO_2_ as standard. IL-1β and TNF-α release were measured with ELISA kits according to the manufacturer’s instructions.

### Co-culture, no contact co-culture of cortical neurons and microglia, and conditioned medium transferring experiment

Cortical neurons were cultured on poly-L-lysine-coated 24-well plates. For the co-culture experiment, neurons were co-cultured with microglia at a cell ratio of 5/1 in Neurobasal medium/B27 supplement after washing with Neurobasal medium/B27 supplement. Cells were incubated for 24 h to verify the direct effect of microglia on cortical neuron viability. For the no contact co-culture experiment, microglia was grown adherent on a coverslip. After wash, the coverslip was up-side down covered on the neuron culture leaving a space/gap between the cells by cushioning three coverslips (2 mm diameter) at the side and then incubated in Neurobasal medium/B27 supplement. To verify the effect of microglia on Aβ-mediated neurotoxicity, the co-culture of neurons and microglia was treated with fAβ25-35 for 24 h. In the conditioned medium transferring experiment, the conditioned neurobasal medium of Aβ-treated microglia was transferred to the neuron culture, in which two cells are not contact or close to each other like that in the “no contact co-culture”.

### Statistic analysis

The results are expressed as the mean ± standard deviation (S.D.) and were analyzed by analysis of variance (ANOVA) with post hoc multiple comparisons corrected with Bonferroni tests.

## Results

### Characterization of microglia phenotype and their inflammatory responses following oAβ1-42 and fAβ25-35 treatment

**Figure 1:**
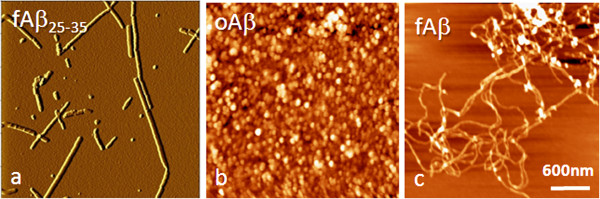
**The structure of the Aβ peptides used in this study.** Aβ25-35 **(a, fAβ**_**25-35**_**)**, oligomeric Aβ1-42 **(b, oAβ)** and fibrillary Aβ1-42 **(c, fAβ)** were spotted on mica and the nano-morphology were observed using atomic force microscopy. The representing images are showed.

To confirm the conformation of the prepared Aβs, the biochemical and morphological nature of fAβ, and oAβ were studied by atomic force microscope (Figure 1).

**Figure 2:**
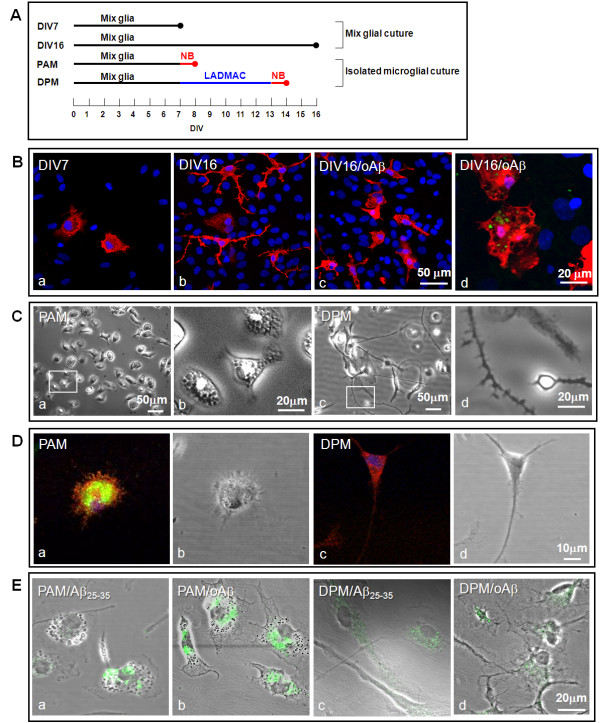
**Microglial culture protocol and Aβ-mediated activation of two phenotypic microglia. A**. The diagram represents the culture protocol for mixed glia and purified microglia. Mixed glia were cultured to DIV 7 and 16. Amoeboid microglia were detached from the mixed glial culture at DIV 7 and cultured in Neurobasal medium (NB) for 24 h (PAM). PAM was further incubated in medium containing the LADMAC-conditioned medium for 6 days and then cultured in NB for 24 h to produce DPM. **B**. Mixed glial cells at DIV 7 and DIV16 (a,b), and mixed glial cells at DIV16 were treated with 5 μM oAβ1-42 for 24 h (c, d). Microglia were immunostained with anti-Iba-1 (red in a, b), anti-CD11b (red in c, d) and anti-Aβ antibodies (green in c, d). Nuclei were stained using Hoechst33258 (blue). **C**. The morphology of PAM (a) and DPM (c) were observed with phase-contrast microscopy. PAM and the spiny processes of DPM indicated in the marked areas were magnified (b, d). **D**. The activation states of PAM (a) and DPM (c) were examined with anti-CD11b (red) and anti-CD68 (green) antibodies. The phase contrast images of each florescent image are presented (b, d). **E**. The representative images show internalized fibrillar Aβ25-35 (a, c) and FITC-conjugated oAβ1-42 (b, d) in both PAM and DPM. Internalized Aβ25-35 was detected with anti-Aβ antibody (green). Each experiment was performed in triplicate.

The mixed glial culture was cultured for 7 and 16 days and the morphology of microglia were determined by Iba-1 immunostaining. The result showed that amoeboid and ramified microglia was observed at 7 and 16 days *in vitro* (DIV), respectively (Figure 2A, B a, b). After oAβ treatment, the processes of ramified microglia (16 DIV) shortened (Figure 2B c), and internalized Aβ was located in the phagocytic vesicles (Figure 2B d). The results suggested that ramified microglia acquired by prolonged incubation were morphologically transformed to an amoeboid phenotype by oAβ.

For the functional study of specific microglia phenotypes, the proliferating amoeboid microglia (PAM) was isolated from the mixed glia at 7 DIV (Figure 2A). PAM possess small cell bodies (diameter: 20–30 μm) with large amounts of phagocytic vacuoles, and some cells have short processes (Figure 2C a, b). To obtain the differentiated microglia, PAM were cultured in medium containing 20% LADMAC-conditioned medium with a high concentration of M-CSF for 5 days to differentiate PAM into process-bearing microglia (DPM). DPM displayed rod-like morphologies and mostly had long processes with branches and spines at one end (Figure 2C c, d). The activation markers CD11b and CD68 were examined to verify their activation states (Figure 2D). The result showed that PAM expressed more CD11b and CD68 than DPM did, indicating that PAM have more phagocytotic activity than DPM. Furthermore, the phagocytic ability of PAM is higher than DPM in response to both fAβ25-35 and oAβ (Figure 2E).

### DPM induce neurotoxicity in co-culture with neurons

**Figure 3:**
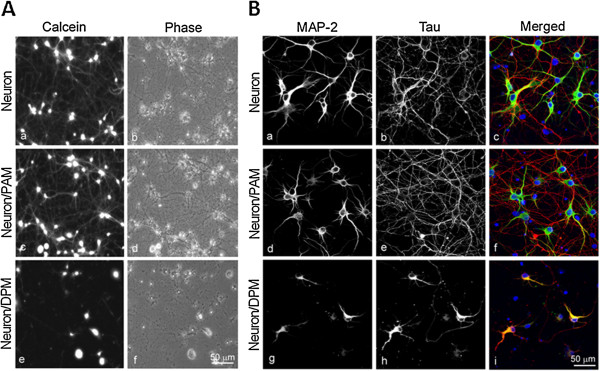
**DPM, but not PAM, is neurotoxic to neuron/microglia co-cultures.** Cortical neurons were cultured alone or with PAM or DPM for 24 h. **A**. The cells were loaded with calcein-AM. The results are shown in representative fluorescence images (a, c, e) and phase contrast images (b, d, f). **B**. Neuronal process integrity was verified with immunostaining for MAP-2 and tau in cortical neurons alone (a-c) or co-cultured with PAM (d-f) or DPM (g-i).

The survival of rat cortical neurons in the presence of PAM or DPM without exogenous stimulation was evaluated using a calcein-AM assay. The co-culture of DPM, but not PAM, with cortical neurons caused neuronal death (Figure 3A). The numbers of axons and dendrites were significantly decreased in cortical neurons co-cultured with DPM, but not PAM, as shown by the immunostaining of tau and MAP-2 to examine the intact of axon and dendrite, respectively (Figure 3B). The results suggested that DPM, but not PAM, injured the co-cultured neurons.

### Aβ-mediated neurotoxicity was diminished by PAM and aggravated by DPM

**Figure 4:**
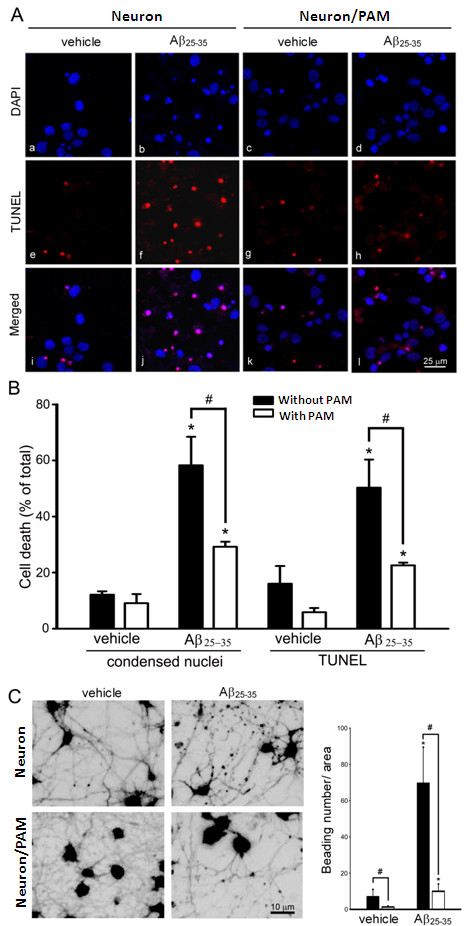
**Aβ-mediated neurotoxicity was attenuated by PAM. A**. Cortical neurons alone or co-cultured with PAM were treated with vehicle or 20 μM Aβ25-35 for 24 h. The nucleus is stained with DAPI (blue) and TUNEL assay (red), and the co-localization of nuclei and TUNEL-positive staining is found in apoptotic cells. **B**. The percentage of apoptosis as determined by DAPI staining (the % of cells with nuclear condensation) and TUNEL assay in cortical neurons alone (closed columns) and co-cultured with PAM (open columns). The results are the mean ± S.D. from four independent experiments. Significant differences between neurons without and with PAM under the same treatments are indicated by #, *P* < 0.05. Significant differences between vehicle-treated neurons and PAM-co-cultured neurons are indicated by *, *P* < 0.05. **C**. After treatment with vehicle or Aβ25-35 for 24 h, the cells were loaded with calcein-AM and confocal images were taken with a confocal fluorescence microscope. The experiment was performed four times. The graph shows the number of beaded neurites per 16 μm^2^ area in neurons alone (closed columns) and neurons co-cultured with PAM (opened columns). The results are the mean ± S.D. from four independent experiments. Significant differences between vehicle-treated cells and Aβ-treated cells co-cultured with microglia are indicated by *, *P* < 0.05. Significant differences between cortical neurons with and without PAM are indicated by #, *P* < 0.05.

Next question we ask is whether PAM can protect neurons against Aβ toxicity. The co-culture experiment permitted contact of microglia and neurons (Figure 4A). The nuclear condensation and apoptosis shown by DAPI staining and TUNEL assays indicate that the fAβ25-35 was neurotoxic, and the co-culture with PAM attenuated both phenomena. The quantification of nuclear condensation and TUNEL-positive signals indicated that fAβ25-35-induced nuclear condensation and apoptosis in cortical neurons were significantly attenuated by co-culturing with PAM (Figure 4B). Furthermore, PAM significantly decreases the number of beaded neurites induced by fAβ suggesting that PAM may also rescue the Aβ-mediated injury of neurites (Figure 4C).

**Figure 5:**
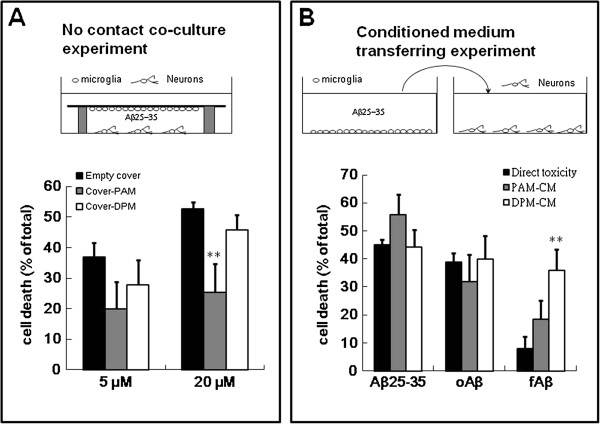
**No contact PAM protects neurons against Aβ25-35-mediated toxicity and the condition medium of DPM aggravated Aβ-mediated neurotoxicity.** The protocol of no contact co-culture experiment and conditioned medium transferring experiment are described in the Methods section and illustrated in the upper panel of **A** and **B**, respectively. **A**. Cortical neurons were co-cultured but not contacted to the coverslip and was not seeded with microglia (black columns), seeded with PAM (gray columns), or seeded with DPM (white column). The cultures were treated with 5 μM or 20 μM fibril Aβ25-35 for 24 h. The coverslip was removed and then the viability of neurons was detected using MTT reduction assay. Results are means ± S.D. from four independent experiments. Significant differences between neurons co-cultured with coverslip seeded and not seeded with microglia are indicated by **, *P* < 0.01. **B.** Cortical neurons were cultured in normal medium (black columns), conditioned medium from Aβ-treated PAM (gray columns) or Aβ-treated DPM (white columns). Three different species of Aβ including fAβ25-35, oAβ, and fAβ were used. Neuronal viability was assessed using an MTT reduction assay. The results are the mean ± S.D. from four independent experiments. Significant differences between neurons and neurons incubated with microglial condition medium are indicated by *, *P* < 0.05.

Since DPM could not be used in the co-culture experiment because of the neurotoxicity may derived from the direct contact with neurons. Therefore, the no contact co-culture experiment was performed (Figure 5A). As the coverslip was not seeded with microglia, fAβ25-35 at 5 and 20 μM induced the cell death by 17.8±9.9% and 43.0±7.9%, respectively. The cell death was reduced only by the coverslip seeding PAM to 25.3±9.5% as the cells treated with 20 μM fAβ25-35.

Aβ activated microglia may secrete some beneficial or destructive factors. Therefore, the effects of the conditioned medium of Aβ-treated PAM and DPM on Aβ-mediated neurotoxicity were examined. The neurotoxicity mediated by Aβ was not reduced by the conditioned medium of Aβ-treated PAM (Figure 5B). The result suggests that the concentration of the PAM derived neurotrophic factor in the conditioned medium may be not high enough to protect neurons against Aβ-mediated neurotoxicity. On the contrary, the concentration of neuron accessible microglia-derived neurotrophic factor is high enough in co-culture or no contact co-culture experiments. The conditioned medium of fAβ-treated DPM significantly induced the neurotoxicity, since the direct neurotoxicity mediated by fAβ alone was marginal (Figure 5B). The results indicated that DPM not only induced the contact neurotoxicity but also mediated the inflammatory neurotoxicity induced by fAβ.

### The pro-inflammatory activation of DPM was more potent than that of PAM

**Figure 6:**
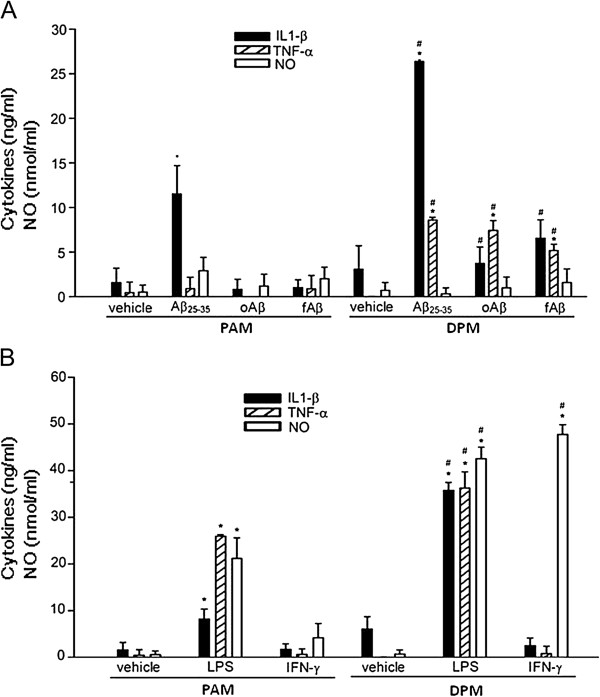
**DPM is more pro-inflammatory than PAM after activated by Aβ, LPS, and IFN-γ. A**. PAM and DPM were treated with vehicle, 20 μM Aβ25-35, 1 μM oAβ or 1 μM fAβ for 24 h. After incubation, cultured medium was collected to determine the level of IL-1β (black columns), TNF-α (striped columns) and NO (white columns). **B**. PAM and DPM were treated with vehicle, 20 ng/ml LPS or 5 ng/ml IFN-γ for 24 h. After incubation, cultured medium was collected to determine the level of IL-1β (black columns), TNF-α (striped columns) and NO (white columns). The results are the mean ± S.D. from six independent experiments. Significant differences between vehicle-treated cells and Aβ-treated cells are indicated by *, *P* < 0.05. Significant differences between PAM and DPM under the same treatments are indicated by #, *P* < 0.05.

The mechanism that DPM aggravating Aβ-mediated neurotoxicity was then studied by determining the release of IL-1β, TNFα and nitric oxide by fAβ25-35-, oAβ-, and fAβ-activated PAM or DPM (Figure 6A). fAβ25-35 induced release of 11.51 ± 3.16 and 26.36 ± 1.07 ng/ml IL-1β in PAM and DPM, respectively. In contrast, oAβ and fAβ did not significantly induce IL-1β release in PAM or DPM. Three Aβs induce TNF-α release by DPM, but not by PAM, and fail to induce nitric oxide release by both DPM and PAM. The results suggest that Aβs were more potent inflammatory stimuli to DPM than that to PAM.

Furthermore, LPS and IFN-γ were employed as the other inflammatory stimulus to examine the inflammatory potency of DPM and PAM. LPS induced release of 35.74 ± 1.71 and 8.18 ± 2.14 ng/ml IL-1β in DPM and PAM, respectively. Moreover, LPS induced release of 36.33 ± 3.44 and 25.91 ± 0.33 ng/ml TNF-α in DPM and PAM, respectively. IFN-γ, however, did not induce release of IL-1β and TNFα in both PAM and DPM (Figure 6B). Furthermore, LPS induced release of 45.8 ± 2.9 and 21.6 ± 7.2 nmol/ml nitric oxide in DPM and PAM, respectively. IFN-γ induced 47.7 ± 2.1 nmol/ml and marginal amount nitric oxide release in DPM and PAM, respectively. The results indicate that DPM are more potent inflammatory mediator than PAM as activated by Aβ, LPS, or IFN-γ.

## Discussion

The increased cell density of microglia due to recruitment and proliferation is common to AD and other neurodegenerative diseases. However, the role of proliferating and differentiated microglia in AD remains unclear. In the present study, we demonstrated that microglia may stay in different activating phenotypes. The proliferating amoeboid microglia (PAM) possesses low inflammatory potency and protects neuron against Aβ-mediated toxicity by phagocytosis and the released neurotrophic factors. DPM is more pro-inflammatory than PAM, and is toxic to the co-cultured neurons directly or indirectly through DPM-secreted toxic factors induced by stimuli, like fAβ. These results provide critical evidence that microglia directly contribute to promoting or opposing neuronal survival in AD.

Microglia have been suggested to help restore normal brain homeostasis by participating in repair and resolution processes after injury [29,43-45]. Part of the resolution process involves the promotion of phagocytosis, reduction of pro-inflammatory mediators, and increased production and release of anti-inflammatory cytokines and cytoactive factors involved in repairing the damaged brain [16]. Nevertheless, few studies have been conducted on the functional characterization of newborn microglia differentiated from proliferating amoeboid microglia in response to Aβ and to determine the roles of microglia in the vicinity of senile plaques. An age-dependent switch of microglial phenotype from phagocytic to classic cytotoxic was observed in APP/PS1 transgenic mice at 18 months old [46]. TNF-α- and iNOS-positive microglia infiltrated into senile plaques in the hippocampus. The authors proposed that oAβ triggers phenotypic switch in cultured microglia. Based on our study of neurotoxicity elicited by DPM derived from proliferating microglia, we proposed that inflammatory microglia phenotypes near AD neuritic plaques may arise from the differentiation of newly proliferating microglia.

Although microglia with amoeboid and ramified morphology (lectin^+^/CD68^+^ and lectin^+^/CD68^-^) have been identified in the human embryonic brain, the functions of these two types of microglia remain unclear [47]. To specifically characterize the functional role of proliferating amoeboid and differentiated ramified microglia, we employed PAM and differentiated DPM using LADMAC-conditioned medium to culture aged microglia that were isolated in this study [48]. Naïve PAM were characterized by the presence of vacuolated cytoplasm and circular shape as earlier reported [19,40]. In contrast, naïve DPM bear long and spiny processes extending a distance greater than three times the cell body diameter [19].

Kuwabara *et al*. have cultured immature and mature microglia and characterized their response to LPS, including the expressions of iNOS, IL-6, and TNF-α [49]. However, the neuroprotective or neurotoxicity of immature and mature microglia was not assessed in their study. To answer this question, we co-cultured microglia and cortical neurons to demonstrate that DPM directly caused neurotoxicity. Conversely, PAM attenuated fAβ25-35-induced apoptosis and neuritic bead formation. PAM was less able than DPM to produce nitric oxide, IL-1β, and TNF-α in response to inflammatory stimuli. IFN-γ-induced nitric oxide release and Aβ-mediated TNF-α release in DPM were markedly elevated compared to PAM. Different maturation stages for pro-inflammatory activation by various endogenous and exogenous stimuli may contribute to the contrasting outcomes in neuron co-cultures. These results reveal that proliferating PAM and differentiated DPM have differential neuroprotective and neurotoxic functions, in spite of their shared cell lineage.

Substantial evidence has demonstrated that Aβ may have indirect neurotoxic effects *via* microglial activation [3,22]. Microglia may also be beneficial in certain circumstances, such as Aβ clearance and neurotrophic factor secretion [37,50-52]. In the present study, DPM had a direct toxic effect on cortical neurons in co-culture experiments in the absence of stimuli. This *in vitro* neurotoxicity of PBM may be due to the postulate disadvantages of PBM culturing [53]. First, PBM is derived from the neonatal brain, which missing the *in vivo* maturation process. Second, PBM are grown in serum- and M-CSF-containing medium, whereas *in vivo* microglia normally never comes in contact with serum components. Third, *in vivo* microglia is kept under constant restraint by variety inhibitory inputs [54] which are not included in the culturing of PBM. Some previous study also indicated the microglia-mediated neurotoxicity in the microglia-neuron co-culture [55,56]. fAβ-mediated neurotoxicity was exacerbated in DPM-conditioned medium. In contrast, PAM attenuated fAβ25-35-mediated neurotoxicity through Aβ phagocytosis and producing neurotrophic factors by the microglia closed enough to the damaged neurons. That was confirmed by the microglia exerting neuroprotective activity in no contact co-culture experiment, but the microglial conditioned medium did protect neurons against Aβ-mediated neurotoxicity. DPM induced pronounced production and release of IL-1β and TNF-α in response to fAβ25-35. The fAβ25-35-mediated production of pro-inflammatory cytokines may reduce the phagocytic response in DPM. Therefore, the elimination of phagocytic capacity during development from neonatal to adult and increased levels of pro-inflammatory cytokines in pathological brain may provide an explanation for why activated microglia are unable to effectively phagocytose fAβ deposits in the AD brain and AD animal models.

## Conclusions

PAM and DPM were cultured to specifically characterize the functional roles of proliferating and differentiated microglia. PAM can be activated and may abrogate Aβ-mediated neurotoxicity by phagocytosing Aβ and the released neurotrophic factors. DPM is a pro-inflammatory phenotype that can mediate neurotoxicity if activated to the classic activation state by LPS, IFN-γ, Aβ or neuronal debris. These results raise the possibility that activation of microglia with a neuroprotective phenotype may have therapeutic potential for treating AD as well as other neurodegenerative conditions.

## Abbreviations

Aβ: Amyloid β protein; AD: Alzheimer’s disease; CNS: Central nervous system; DPM: Differentiated process-bearing microglia; DIV: Days in vitro; FBS: Fetal bovine serum; IFN-γ: Interferon-γ; iNOS: Induced nitric oxide synthase; IL-1β: Interleukin-1β; LPS: Lipopolysaccharide; M-CSF: Macrophage-colony stimulating factor; MAP2: Microtubule associated protein 2; MEM: Minimum essential medium; PAM: Proliferating amoeboid microglia; TNF-α: Tumor necrotic factor-α.

## Competing interests

The authors declare that they have no competing interests.

## Authors’ contributions

YCH and HJT participated in the design of the study and performed the statistical analysis. CPC, YCT and YHW carried out the cell culture and immunoassays. MFW and FYC participated in amyloid β protein preparation and neurotoxicity assay. FLH participated in figure editing. YJS participated in its design and coordination and helped to draft the manuscript. All authors read and approved the final manuscript.
